# The remarkable potential of machine learning algorithms in estimating water permeability of concrete incorporating nano natural pozzolana

**DOI:** 10.1038/s41598-024-62020-3

**Published:** 2024-05-31

**Authors:** Shtwai Alsubai, Abdullah Alqahtani, Sabih Hashim Muhodir, Abed Alanazi, Mohd Ahmed, Dheyaa J. Jasim, Sivaprakasam Palani

**Affiliations:** 1 Department of Computer Science, College of Computer Engineering and Sciences in Al-Kharj, Prince Sattam Bin Abdulaziz University, P.O. Box 151, Al-Kharj 11942, Saudi Arabia; 2 Department of Architectural Engineering, Cihan University-Erbil, Kurdistan Region, Iraq; 3 Department of Civil Engineering, College of Engineering, King Khalid University, PO Box 394, Abha 61411, Saudi Arabia; 4 Center for Engineering and Technology Innovations, King Khalid University, Abha 61421, Saudi Arabia; 5 Department of Petroleum Engineering, Al-Amarah University College, Maysan, Iraq; 6 College of Electrical and Mechanical Engineering, Addis Ababa Science and Technology University, P.O.Box 16417, Addis Ababa, Ethiopia

**Keywords:** Reinforced concrete, Nano natural pozzolana, Water penetration depth, Machine learning, Laboratory tests, Civil engineering, Composites

## Abstract

This paper aims to estimate the permeability of concrete by replacing the laboratory tests with robust machine learning (ML)-based models. For this purpose, the potential of twelve well-known ML techniques was investigated in estimating the water penetration depth (WPD) of nano natural pozzolana (NNP)-reinforced concrete based on 840 data points. The preparation of concrete specimens was based on the different combinations of NNP content, water-to-cement (W/C) ratio, median particle size (MPS) of NNP, and curing time (CT). Comparing the results estimated by the ML models with the laboratory results revealed that the hist-gradient boosting regressor (HGBR) and K-nearest neighbors (KNN) algorithms were the most and least robust models to estimate the WPD of NNP-reinforced concrete, respectively. Both laboratory and ML results showed that the WPD of NNP-reinforced concrete decreased with the increase of the NNP content from 1 to 4%, the decrease of the W/C ratio and the MPS, and the increase of the CT. To further aid in the estimation of concrete’s WPD for engineering challenges, a graphical user interface for the ML-based models was developed. Proposing such a model may be effectively employed in the management of concrete quality.

## Introduction

A concrete structure's performance and durability must be adequate for its intended usage environment and expected lifespan^1–5^. Concrete with these qualities is said to be long-lasting or stable^6–10^. The concrete's inability to withstand wear and tear might result from both internal and external influences^11^. When concrete is completely dry, its permeability increases above its value under normal circumstances. When the proportion of water to cement (W/C) ratio rises, so does the air permeability factor. Yet, concrete mixes are known to exhibit strange characteristics^12,13^. While increasing the concrete's permeability by adding air has little effect on water absorption, it does decrease the concrete's strength, which in turn increases the concrete's permeability^3,4^. However, less permeable concrete can be achieved in light concrete by substituting more compact materials^14^.

Experiments conducted on concrete specimens including various nanomaterials and kinds of fly ash show that the water permeability of concrete is decreased due to the addition of these components. The abrasion and compressive strength of concrete specimens made with several percentages of ash improved with curing time, with the greatest result being achieved at 15% ash, while the permeability typically decreased with the addition of ash at every percentage^6–8^. Nano-capacity silica's ability to absorb Ca(OH)_2_ crystals and reduce their quantity results in a more compact and dense region being generated in the specimens produced by nano-silica, which in turn increases the mechanical strength of the concrete and its longevity. This reduces the concrete's permeability^15^. Concrete's compressive strength to the infiltration of fluids like water, carbon dioxide, and oxygen is reflected in its permeability, which is thus an important indicator of the material's durability^10,11^.

The engineering community is finding more and more uses for nanoparticles such as natural pozzolana (NP) in the concrete’s mixture^16^. Cement substitution is one of its most prevalent uses^17^. The use of NP in the composition of concrete significantly reduces the permeability of water. There are also environmental, financial, and technological advantages to using this application^18^. Compared to the cement industry, which accounts for around seven percent of global CO_2_ emissions, the amount of CO_2_ emitted during its processing is rather little^19,20^. Also, the energy used to produce NP is much lower than that used to produce ordinary Portland cement (OPC). This kind of pozzolana is already organically burned, so it doesn't need to be added to the kiln. In addition, it enhances the performance of concrete by enhancing certain qualities. Consequently, NP may be used as a cement alternative, resulting in greener concrete^21^. Yet, poor early strength concrete is one of its drawbacks when applied at the micro level. Grinding NP to a micro size is one of the most recent approaches taken to fix this problem^16^. In addition to their filling effects, NP nanoparticles may speed up cement hydration^22^.

Depending on the type of concrete required, different permeabilities are required in the designs for the concrete mixture. To produce such concrete, several laboratory tests are conducted on the concrete samples made of different compounds. Finally, the best mix for the desired concrete is obtained. However, laboratory tests to determine concrete's water permeability are expensive, time-consuming, and labor-intensive. In addition, there is always some degree of ambiguity in laboratory findings due to human error and different laboratory circumstances^16^. As a result, it is crucial to have an accurate estimation tool for estimating the water permeability when developing concrete mixes for construction projects. For this purpose, providing models based on machine learning (ML) methods can be a suitable option^16^.

ML has found extensive application across diverse domains within civil engineering, including the realm of building materials, where it aids in mix design and optimization of cement material performance^23,24^. ML models excel particularly in handling large datasets, showcasing their versatility across various data types^25,26^. With their remarkable capacity to discern intricate and unpredictable correlations among input and output variables in datasets, ML systems have witnessed significant utilization in addressing practical challenges^27^. This surge in their usage underscores their effectiveness in unraveling complex relationships and patterns within datasets, thereby contributing to advancements in solving real-world engineering problems.

Only the research by al-Swaidani et al.^16^ concentrated on the use of ML approaches for estimating the water permeability of nano natural pozzolana (NNP)-reinforced concrete, even though many other studies have focused on laboratory testing of NP-reinforced concrete^28,29^. Using data obtained during an experiment, they estimated the water penetration depth (WPD) of NNP-reinforced concrete using a trifecta of ML techniques: multiple linear regression (MLR), artificial neural network (ANN), and fuzzy logic (FL). They arrived at the conclusion that the ANN and FL methods are useful for estimating the WPD of NNP-reinforced concrete.

The ongoing study has exerted considerable effort employing a variety of widely recognized ML techniques to generate exceptionally precise models for estimating the WPD of NNP-reinforced concrete. Due to their advantageous features and merits, scholars have embraced these algorithms for utilization across a diverse array of projects. Here's a concise overview of these benefits:

- They possess the capability to handle both linear and nonlinear data for tasks involving classification and regression.
- Utilizing a decision boundary in the form of a hyperplane, node, or neuron enables effective differentiation between groups.
- Simultaneously presenting options and choices while considering costs and potential benefits facilitates the evaluation of capabilities and errors.
- There is a notable reduction in computation time, enhancing the potential for utilization across all phases of assessment and stabilization; the accuracy of calculations and errors can be gauged relative to the study's complexity.
- Enhanced precision in forecasting is facilitated, leading to a decrease in errors.
- Their usage is characterized by simplicity and effectiveness.

The ML models are constructed based on 840 data points recorded for 840 cube-shaped concrete specimens with dimensions of 15 × 15 × 15 cm in the laboratory tests. The preparation of concrete specimens is based on the different combinations of NNP content (0, 1, 2, 3, 4 and 5%), W/C ratio (0.1, 0.2, 0.3, 0.4, 0.5, 0.6, 0.7, and 0.8), median particle size (MPS) of NNP (100, 200, 300, 400, and 500 nm), and curing time (CT = 2, 7, 28, 90, and 180 days). Therefore, these four parameters are considered as the inputs of the ML models. At long last, through the meticulous comparison of ML model outcomes with laboratory findings, each model's efficacy in estimating the WPD of NNP-reinforced concrete is scrutinized, pinpointing the most fitting model.

The noteworthy contributions of this endeavor encompass:

- Employing a robust database derived from a validated laboratory examination.
- Delving into the performance nuances of 12 distinct ML methodologies in WPD estimation for reinforced concrete.
- Precision-tuning the hyperparameters of each model to attain optimal estimations.
- Conducting a comprehensive analysis of both laboratory-derived and ML-generated results, juxtaposing their findings.
- Evaluating the models' efficacy against real-world scenarios.
- Conducting a thorough sensitivity analysis of input parameters.
- Presenting the most potent ML-driven model for WPD estimation in NNP-reinforced concrete.
- Introducing a user-friendly graphical interface (GUI) founded on ML models for WPD estimation in NNP-reinforced concrete.

Proposing such a model may be effectively employed in the management of concrete quality because it may give fresh information or experiences that contribute to a better understanding of the complex nature of the interaction between the ingredients and the qualities of NNP-reinforced concrete. In addition, the site engineers may benefit from such knowledge or experience when attempting to estimate the concrete's quality in advance of placement based on the mix’s components, the amount of time required to open the formwork, and other factors unique to the project.

**Figure 1:**
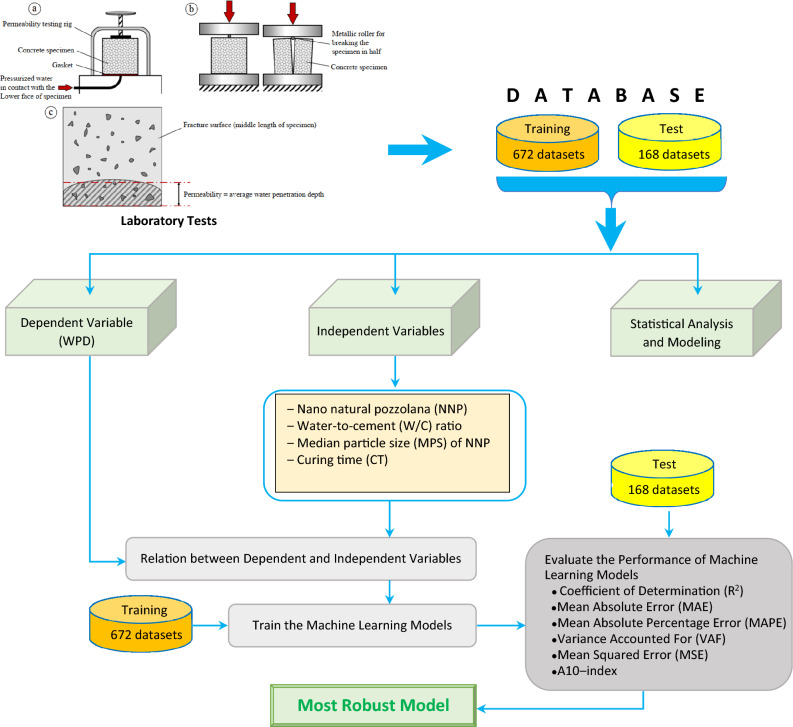
The whole process of this study.

The whole process of this study is shown in Fig. 1.

## Machine learning methods

In this section, several supervised ML techniques are discussed, including ANN, GPR, SVR, RF, KNN, MLPR, XGBoost, GBR, VR, HGBR, ETR, and DTR. The purpose of supervised learning is to learn how to assign labels to data by training a mapping function. It is given a dataset of training examples that have been labeled. It's possible to classify them as either "classification" or "regression" issues. Regression problems are supervised learning tasks in which the goal variables are numeric, while classification issues are those in which the target variables are a set of labels^30^.

ANNs are computational systems inspired by the intricate neural networks found in living organisms. At the core of an ANN lies a network comprised of interconnected nodes, known as artificial neurons, designed to mimic the functionality of biological neurons in the brain^31^. Similar to the synapses found within a living brain, these connections facilitate the transmission of information between neurons. An artificial neuron is capable of processing input received from other neurons and emitting its signals. Each neuron's output results from the application of a non-linear function to the summation of its inputs, wherein the "signal" conveyed through a connection is denoted by a real number. These connections are depicted as edges. Throughout the learning journey, the importance of neurons and edges transforms. The intensity of the signal relayed through a connection is contingent upon its weight. Neurons may possess a threshold, whereby they only emit a signal if the cumulative input surpasses a certain level. Neurons often organize into layers, with inputs undergoing distinct modifications at various levels. Signals may traverse multiple layers iteratively on their journey from the input layer to the output layer^32^. MLPR represents a type of ANN model designed to enhance prediction accuracy through the utilization of backpropagation to refine the weights assigned to connections among neurons. Within MLPR, the implementation of a multi-layer perceptron algorithm for testing and training datasets involves employing approaches such as stochastic gradient descent and backpropagation^33^.

To enhance prediction accuracy and mitigate overfitting, ETR employs an averaging technique involving fitting multiple random decision trees to diverse subsamples of the dataset^31^. GBR is very effective in resolving regression issues. The gradient amplification model takes a set of weak starting models and progressively merges them into one robust end model. Belonging to the cohort of learning algorithms, this method has consistently demonstrated the effectiveness of a straightforward or elementary algorithm. Nonetheless, this verdict is highly contingent upon the attributes of the provided data. Each tree node's output value is determined using the following formula with a probability of 0.5 as the initial guess^15,17,18,21,34^.

To expedite the tree-learning process, HGBR may be used to discretize continuous variables. XGBoost employs a 0.5 probability to estimate the final output value. The regularization parameter in GBR and XGBoost differs in how it adjusts the modest effects of very large leaves^35^.

Once a dataset has been linearly categorized, SVR selects the optimal route through quadratic programming. GPR has been causing a stir in the realm of machine learning by adopting a Bayesian and nonparametric approach to regression. Among its myriad benefits, GPR stands out for its ability to provide uncertainty estimates for predictions and its robust performance on small datasets. Meanwhile, the DTR technique constructs regression trees by iteratively generating a decision tree for each subset of the dataset^36^. Some nodes will represent options, and others will represent leaves in a tree structure^37^. Classification and regression are only two examples of the types of issues that may be tackled with the help of RF, an ensemble learning technique. During training, it produces a large number of decision trees, all of which are important. The result of a regression operation is the mean forecast of all trees^38^.

To get precise estimates of the relevant parameters, VR belongs to a class of ensemble meta-estimators that use several base regressors. The final forecast is the mean of many different predictions^39^. In the KNN approach, the distance between two points can serve as a decisive factor, especially when it exceeds a certain threshold. Weight assignment to each data point hinges on its alignment with the initial training data^15,40^.

## Dataset preparation

### Samples' preparation and experimental test

The studied dataset was constructed using the authors' experimental findings. 840 cube-shaped concrete samples with dimensions of 15 × 15 × 15 cm were prepared. Concrete mixtures were prepared with six nano natural pozzolana (NNP) contents (0, 1, 2, 3, 4, and 5%), eight water/cement (W/C) ratios (0.1, 0.2, 0.3, 0.4, 0.5, 0.6, 0.7, and 0.8), five particle sizes of NNP (100, 200, 300, 400, and 500 nm), and five curing times (CT = 2, 7, 28, 90, and 180 days). The concrete mixes were developed in a manner that was compliant with the recommendations that were presented in ACI 211. The investigated NP was obtained from a quarry that is situated in the northwestern part of Iran. The main oxides of the investigated NP are SiO_2_ (41%), Al_2_O_3_ (22%), Fe_2_O_3_ (15%), MgO (12%), CaO (5%), Na_2_O (3%) and K_2_O (2%). The NP was crushed down to the examined sizes of 100 nm, 200 nm, 300 nm, 40 nm, and 500 nm using a laboratory centrifugal ball mill (Retsch, S100, Germany) for a total of 360 min, 335 min, 314 min, 293 min, and 275 min, respectively. The ratio of NP dispatch to steel balls that was ultimately decided upon was 1/5 at a 300-rpm revolution number.

Crushed dolomitic rock from nearby quarries was utilized for aggregates in concrete, with fine aggregate having SG = 2.78 & absorption = 1.63%, medium aggregate having SG = 2.81 and absorption = 1.35%, & Los Angeles Nr. = 20.2, and coarse aggregate having comparable physical qualities to the latter. To get the aggregate mix closer to Fuller's grading, it was mixed in some natural river sand (SG = 2.65 & absorption = 1.58%).

The cement for the experiment came from a nearby cement factory. This material has a median particle size (MPS) of 17 µm and a Blaine fineness of 3500 cm^2^/g. C_3_S (65%), C_2_S (18%), C_3_A (12%), and C_4_AF (5%), are all possible minerals that might make up this material. It took 181 min to set up initially and 245 min to finish. A consistent ratio of coarse aggregate to total aggregate was maintained throughout the development of all concrete compositions. All combinations had the same cement proportions, or 350 kg of cement per cubic meter. In the experimental section, the mixing procedure described in al-Swaidani et al.^16^ was adopted.

**Figure 2:**
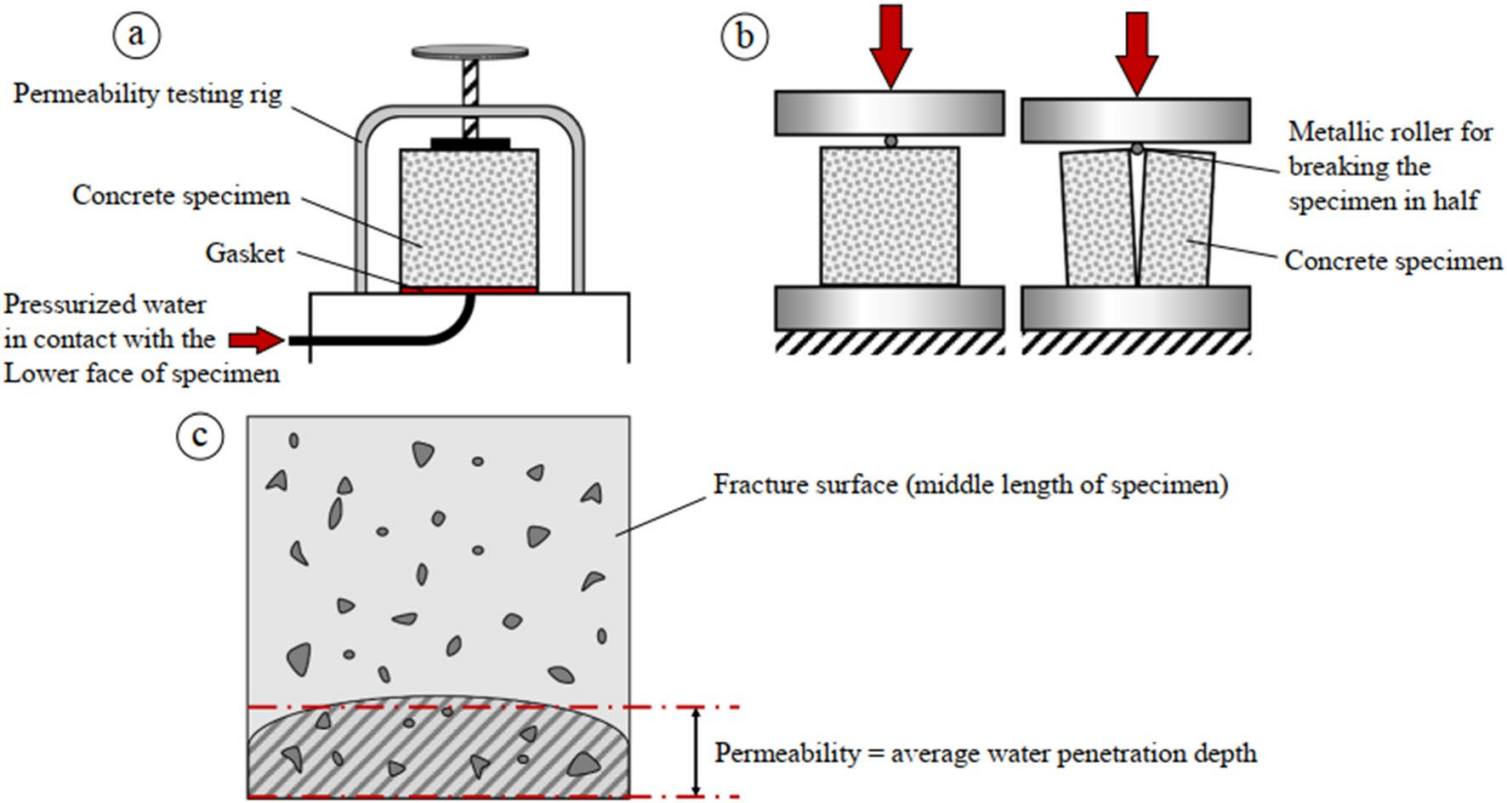
A simplified diagram showing the steps involved in a permeability test: (a) placing a concrete specimen under pressure in a water bath; (b) cracking the specimen in half to measure its permeability; and (c) measuring the mean length of the wet concrete at the fracture^14^.

The hydraulic conductivity of a material is its capacity to allow fluid to flow through its pores in response to a pressure gradient. The concrete's permeability coefficient provides insight into the material's underlying structure and overall quality. The DIN 1048 standard specifies a procedure for measuring the water penetration depth (WPD) in concrete samples subjected to continuous pressure of water. Figure 2 shows a simplified diagram of the permeability testing process. The depth to which the pressurized water has penetrated the specimens is determined by this experiment. These specimens need to be kept between 20 and 25 degrees to cure and be preserved properly. Each specimen is removed from the water and allowed to cure at room temperature for 24 h. The cube specimens are then placed in the apparatus, and a pressure of 5 bars is applied to the top of the specimens to ensure that water can only enter via the objects' surfaces. There is a three-day inspection period stipulated by the standard for the samples. The WPD level in a concrete cube specimen is obtained by breaking the specimen after applying pressure for a predetermined amount of time^14^.

### Statistical analysis of the dataset

**Figure 3:**
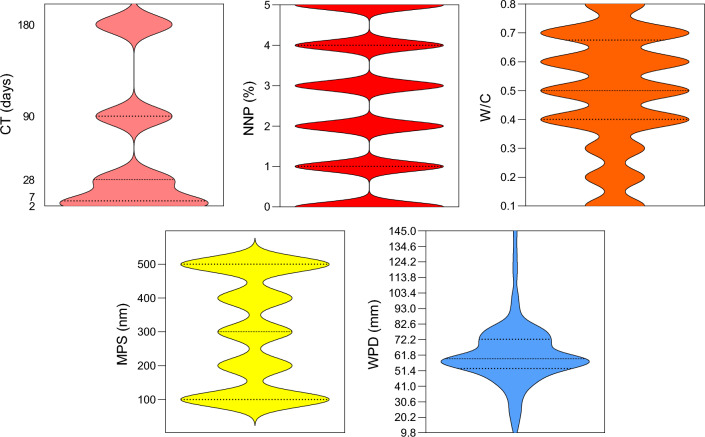
The violin plots of input and output parameters.

In total, the preparation of 840 concrete specimens with different mixtures and the permeability test on each of them took about one year. Finally, 840 datasets including four input parameters (CT, MPS, NNP, and W/C) and one output (WDP) were obtained for use in the ML algorithms. A visual summary of the data’s distribution, showing the median, interquartile range, and density of the data at different values is shown in Fig. 3 through violin plots.

The selection of the four input parameters was driven by the following considerations:

- *Relevance and impact on permeability:* The chosen variables are among the most significant factors affecting concrete permeability^14^. This decision was underpinned by an extensive review of the literature and empirical studies indicating that these variables directly influence the water penetration depth in concrete^14,15,40^. By focusing on these key factors, this research aimed to ensure that the models capture the most critical aspects influencing permeability.
- *Data availability and consistency: *Ensuring a large, consistent dataset is fundamental for training robust ML models. The chosen variables are not only crucial but also ones for which data could be systematically collected and standardized across 840 data points. This consistency is vital for minimizing noise and ensuring the reliability of the model predictions.
- *Model complexity and overfitting:* Introducing a larger number of variables into a machine learning model increases the complexity and the risk of overfitting, especially when dealing with a finite dataset. Overfitting arises when a model becomes overly attuned to the intricacies and irrelevant fluctuations present in the training data, leading to a detrimental effect on its ability to generalize and perform well on unseen data^41^. By limiting the number of variables, this research aimed to strike a balance between model complexity and the risk of overfitting, thereby enhancing the model's generalizability and predictive performance on unseen data.
- *Practical applicability and computational efficiency:* Our objective was to develop a model that is not only accurate but also practical for real-world applications. Models that require a large number of input variables may pose challenges in terms of data collection and computational resources, potentially limiting their utility in practical engineering contexts. The selected variables are readily assessable in the design and construction phases, making our model more accessible to practitioners.

The obtained dataset was split into two parts: a training set (consisting of 80% of the data) and a testing set (consisting of 20% of the data). Therefore, this study employs a database with four-dimensional input training vectors (1 × 6) and one-dimensional output training vectors (1 × 1). Table 1 provides a concise summary of the available dataset. Each parameter's mean, standard deviation (STD), minimum, maximum, and first, second, and third quartiles (1st, 2nd, 3rd) are shown in this table.

**Table 1:** A summary of the database used in this study.

	CT [days]	NNP [%]	MPS [nm]	W/C	WPD [mm]
Training datasets					
Count	672	672	672	672	672
Mean	61.93	2.52	302.08	0.50	62.81
Std	67.44	1.70	160.56	0.19	21.35
Min	2.00	0.00	100.00	0.10	9.80
25%	7.00	1.00	100.00	0.40	53.08
50%	28.00	3.00	300.00	0.50	59.25
75%	90.00	4.00	500.00	0.70	72.45
Max	180.00	5.00	500.00	0.80	145.0
Testing datasets					
Count	168	168	168	168	168
Mean	59.30	2.40	291.67	0.48	62.27
Std	65.88	1.74	160.25	0.20	19.91
Min	2.00	0.00	100.00	0.10	13.00
25%	7.00	1.00	100.00	0.38	52.55
50%	28.00	2.00	300.00	0.50	59.55
75%	90.00	4.00	500.00	0.60	70.25
Max	180.00	5.00	500.00	0.80	140.7

As the first step in data preprocessing, the considered features (CT, NNP, MPS, and W/C) were normalized using the min‒max normalization method. Min–max normalization is a scaling technique used to transform the features of a dataset so that they fall within a specified range (typically 0 to 1) using Eq. 1. This method is particularly useful in ML and data mining where the algorithm's performance might be negatively impacted by the scale of the data.

1 $${x}{\prime}=\frac{x-{\text{min}}(x)}{{\text{max}}\left(x\right)-{\text{min}}(x)}$$

where ${x}{\prime}$ and $x$ are the normalized and original values, respectively. ${\text{min}}(x)$ and ${\text{max}}(x)$ are the minimum and maximum values of the feature ($x$) across all data points.

**Figure 4:**
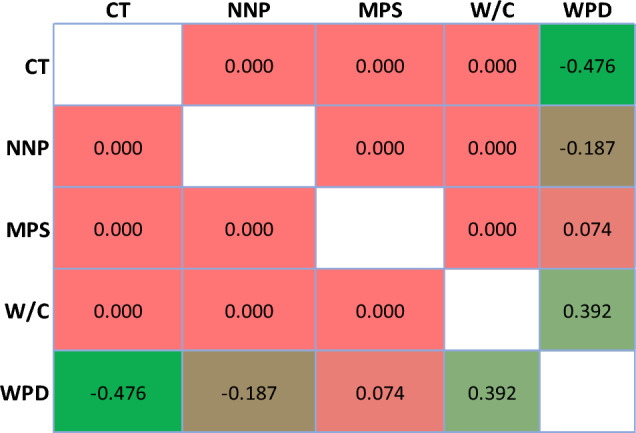
The Pearson correlation matrix between input and output parameters.

After the normalization step, the Pearson correlation matrix depicting the relationship between input and output parameters is shown in Fig. 4. The stark revelation is that the input parameters stand utterly independent of each other, devoid of any discernible relationship. Hence, it's imperative to consider all these parameters. Conversely, the correlation between input and output parameters surpasses 0.07, signifying a clear dependency of all considered features on the output (WPD) parameter. Consequently, they warrant thorough consideration in modeling endeavors.

**Figure 5:**
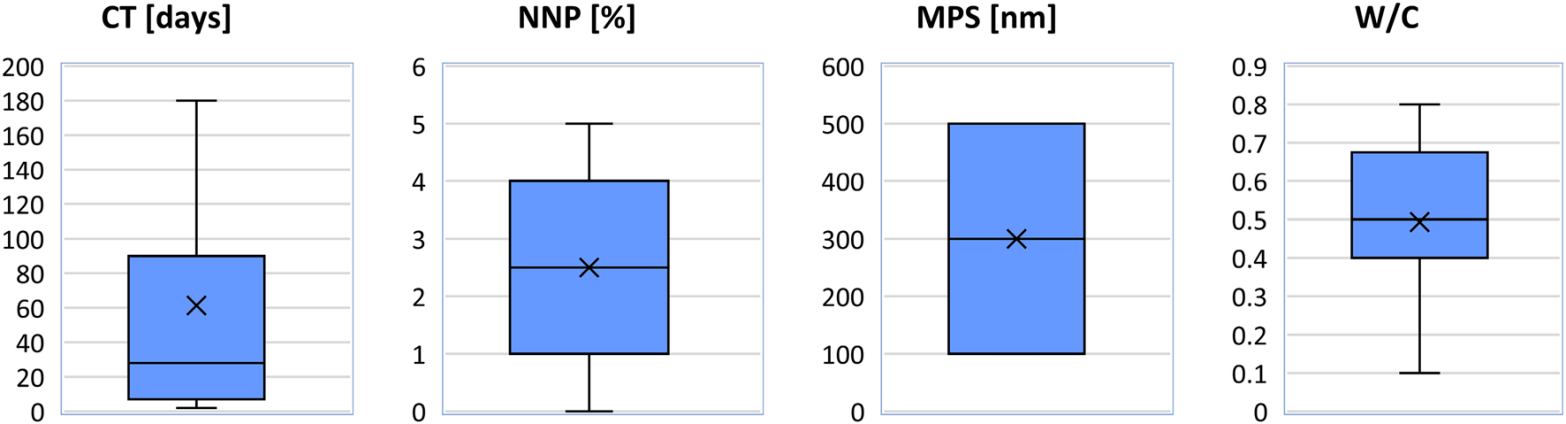
Boxplots of the dataset.

In the next step, descriptive statistics were run on the primary datasets. Figure 5 shows boxplots for all of the parameters. At a glance, it is apparent that parameters NNP and MPS are symmetrical since the median line is located in the middle of the box plots for them. The parameters CT and W/C are not symmetrical. Another critical issue that must be accounted for when processing data is the presence of outlier values for a parameter. Outliers make it more difficult to identify patterns in data collection. Our ability to create reliable forecasting models is enhanced by the removal of anomalous information such as outliers and clusters. As can be observed in Fig. 5, there is no outlier for any of the parameters. The main reason for the absence of outliers is due to considering the following strategies during laboratory tests:

- *Rigorous quality control:* Strict quality control measures were implemented at all stages of the laboratory process, from sample collection to data analysis. This included regular calibration of equipment, standardized procedures for experiments, and thorough training for personnel.
- *Replication of experiments:* More of the experiments were conducted multiple times under the same conditions to confirm the consistency of results. Replication helps identify anomalies that could be outliers due to experimental error.
- *Robust experimental design:* The experiments were designed in a way that minimized the impact of variability. This involved controlling environmental conditions closely and using techniques that were less sensitive to external fluctuations.
- *Pre-experimental data screening:* Pre-experimental screening techniques were employed to identify and exclude potential sources of outliers. For example, samples were checked for contamination or damage before analysis, which could prevent abnormal results.

**Figure 6:**
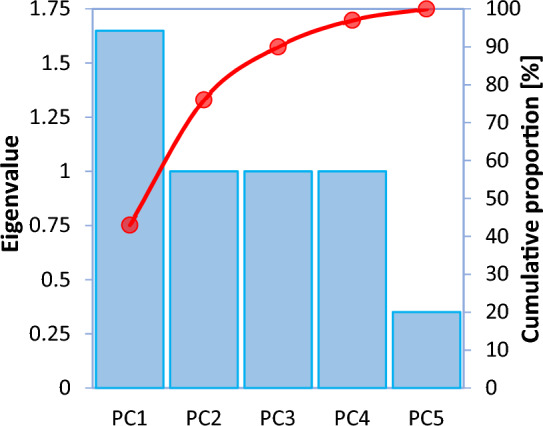
Scree plot for the first four PCs.

**Figure 7:**
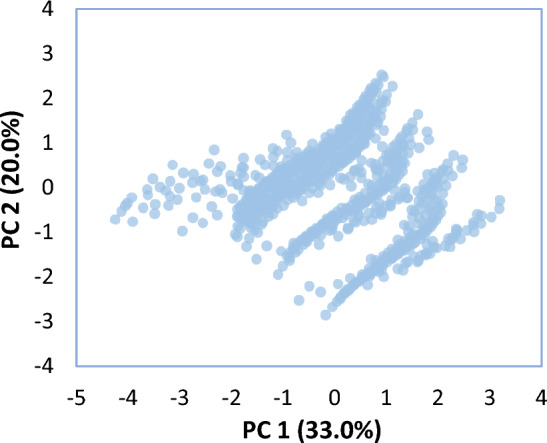
Scatter plot of the PC1 against the PC2.

For feature selection, the principal component analysis (PCA) method was employed. The multivariate statistical technique of principal component analysis may be used to investigate discrepancies and identify trends. Moreover, this method might be used to reduce the number of dimensions in difficult issues (the number of independent variables). In the PCA, the principal components (PCs) which are linear combinations of the original variables, are used to best explain the differences across datasets. Table 2 displays the coefficients (eigenvalues) and percentage of variation of the PCs. To determine which PCs best describe the variability in a dataset, scree charts may be quite helpful (see Fig. 6). The top two PCs explain 76% of the total variance in the data, as seen by the scree plot. Figure 7 displays the scatter plot for the first two PCs. According to this figure, there is no definite natural group and outlier in the dataset.

**Table 2:** Coefficients for the PCs.

Variables	PCs				
	PC1	PC2	PC3	PC4	PC5
CT	0.519	-0.679	0.000	0.000	0.519
NVS	0.203	0.311	−0.899	−0.108	0.203
MPS	−0.080	−0.123	0.040	−0.985	−0.080
W/C	−0.427	−0.654	−0.435	0.134	−0.427
WPD	−0.707	0.000	0.000	0.000	0.707
Eigenvalue	1.649	1.000	1.000	1.000	0.351
Variability [%]	43.000	33.000	14.000	7.000	3.000
Cumulative proportion [%]	43	76	90	97	100

## ML models’ implementation

To implement the ML models, the Jupyter Notebook environment offered in the Anaconda Navigator version 3.7 was utilized. The Anaconda program is a free and open-source scientific computing in Python, which aimed at simplifying package administration and execution. The calculation was carried out on an Intel (R) Core (TM) i7-10750H CPU operating at 2.60 GHz with 32 GB of RAM.

All the ML models were optimized based on the training datasets to show the highest performance in the WDP estimation. To optimize each of the models, the type or value of their hyperparameters must be set to an optimal state. In this study, the trial-and-error method was used to fine-tune the model’s hyperparameters. Finding solutions to challenging problems frequently involves going through a process of trial and error. The agent will try a wide variety of different strategies until they either give up or succeed in accomplishing their goal. This issue will go through a significant number of iterations before the solution that works is discovered. In Table 3, the tuned parameters and hyperparameters for each ML technique are presented.

**Table 3:** Optimized parameters and hyperparameters of the ML models.

Method	Parameters/hyperparameters				
DTR	criterion **= **'squared_error'	random_state **=** 2			
KNN	n_neighbors **=** 6				
SVR	kernel **=** 'rbf'	C **=** 100	epsilon **=** 2	gamma = 1.6	
GPR	seed **=** 0	random_state **=** 5	alpha = 0.9		
	kernel = ConstantKernel(constant_value = 1.0, constant_value_bounds = (0, 17)) * RBF(length_scale = 1, length_scale_bounds = (0, 5)) + RBF(length_scale = 1, length_scale_bounds = (0, 30))				
XGBoost	cv **=** 5	verbose **=** 1	colsample_bytree = 0.5	n_estimators = 100	
	max_depth = 20	estimator = gbm	scoring = neg_mean_squared_error		
RF	estimator = rf	n_iter **=** 100	cv **=** 3, verbose **=** 2	random_state **=** 42	n_jobs **=** **-**1
ETR	Bootstrap = False	oob_score = False	min_samples_leaf = 1		min_samples_split = 4
	criterion = mse	max_depth = 20	max_features = 4		warm_start = True
	n_estimators = 12	random_state = 0			
HGBR	loss = squared_error	learning_rate = 0.1	max_iter=200		max_leaf_nodes = 30
	max_depth = None	min_samples_leaf = 10	l2_regularization = 0		max_bins = 100
	scoring = loss	validation_fraction = 0.1	n_iter_no_change = 5		tol = 1e-08
	verbose = 0	random_state = None			
GBR	loss = squared_error	learning_rate = 0.1	max_leaf_nodes = 30		max_depth = None
	min_samples_leaf = 5	warm_start = False	validation_fraction = 0.1		n_iter_no_change = 5
	tol = 1e-08	verbose = 0	random_state = None		
ANN	#First Hidden Layer	units = 64, activation = relu			
	# Second Hidden Layer	units = 64, activation = relu			
	#Third Hidden Layer	units = 64, activation = relu			
	# Output Layer	units = 1, activation = relu			
	optimizer = adam	loss = mse	batch_size = 8		epochs = 100
VR	n_estimators **=** 6	random_state **=** 2	lr **=** 'drop'		
MLPR	solver = adam	activation = relu	hidden_layer_sizes = 200		alpha = 0.0001
	batch_size = 10	learning_rate = constant	learning_rate_init = 0.1		power_t = 0.
	max_iter = 200	tol = 0.0001	momentum = 0.9		epsilon = 1e-07
	beta_1 = 0.9	beta_2 = 0.999	validation_fraction = 0.1		max_fun = 15000
	n_iter_no_change = 10				

## Model validation

To evaluate the potential of the ML models in the prediction of SFRS, their results were compared with the results estimated using the LEMs and GeoStudio commercial software through five statistical evaluation metrics including the coefficient of determination (R^2^), the mean absolute error (MAE), the mean squared error (MSE), the mean absolute percentage error (MAPE), and the a10_index (Eqs. 2–6).

2 $${R}^{2}=1-\frac{sum\, squared\, regression}{sum\, of\, squares\, total}$$

3 $${\text{MAE}}=\left(\frac{1}{n}\right)\sum_{i=1}^{n}\left|{M}_{i}-{F}_{i}\right|$$

4 $${\text{MSE}}=\left(\frac{1}{n}\right)\sum_{i=1}^{n}{\left({M}_{i}-{F}_{i}\right)}^{2}$$

5 $${\text{MAPE}}=\frac{100\%}{n}\sum_{i=1}^{n}\left|\frac{{M}_{i}-{F}_{i}}{{M}_{i}}\right|$$

6 $${a}^{10}\_index=\frac{m10}{n}$$

where ${F}_{i}$ and ${M}_{i}$ denote the forecasted and measured values, respectively; $n$ is the number of samples; and $m10$ is the number of samples having *experimental/forecasted* ratios between 0.9 and 1.10.

## Results and discussion

### Experimental results

**Figure 8:**
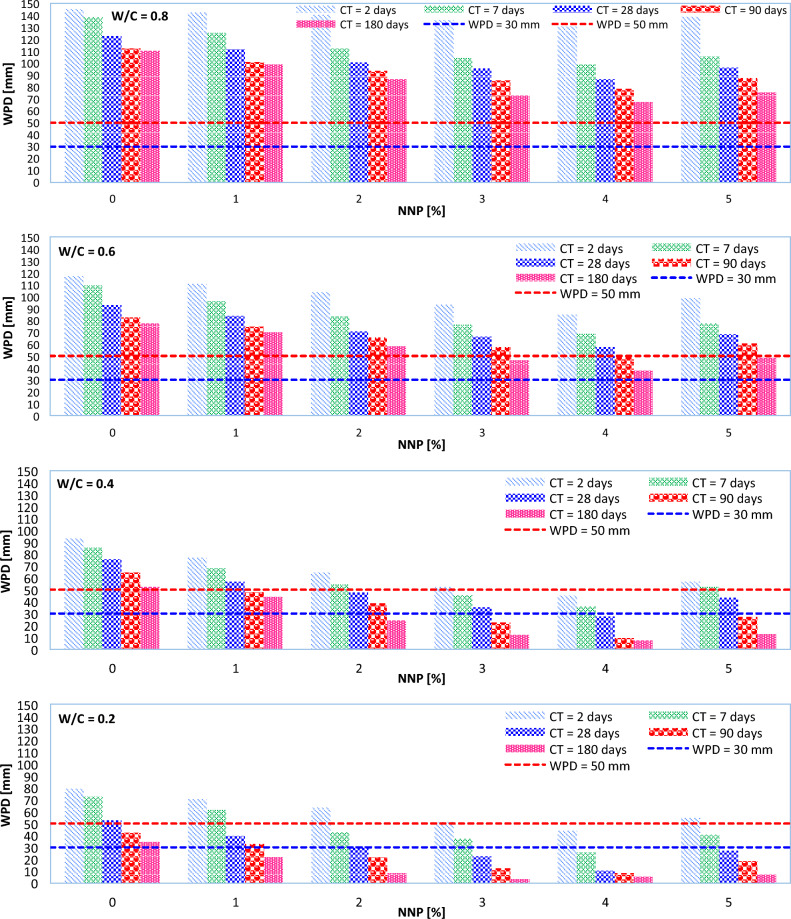
WPD at different CTs and different W/C ratios of 0.2, 0.4, 0.6, and 0.8 (MPS = 500 nm).

The WPD values for the NNP-reinforced concrete samples are shown in Fig. 8 (MPS = 500 nm). A WPD of less than 50 mm classifies a concrete mix as impermeable, and a WPD of less than 30 mm classifies concrete as impermeable even under corrosive conditions^16^. As can be seen in Fig. 8, no W/C = 0.8 concretes were found to be impermeable even after extended curing durations (90 or 180 days). Even after curing for 180 days, no concretes with a W/C of 0.6 were found to be impermeable under corrosive conditions. Impermeable concretes may be made in the early curing days with W/C ratios (0.4 and 0.2).

As can be seen in Fig. 8, when NNP is dosed at above 2% and W/C = 0.4 is used to make the concrete mixes, impermeable concrete is formed after 7 days of curing. Also, these concrete mixes may have WPDs of less than 30 mm after curing for 28 days. Pozzolanic reactions, which produce more cementing compounds like C-A-S–H and C-S–H, may be responsible for the lower WPD^12,14^. NNPs, because of their size, also have a filling effect, which makes the resulting concrete denser and less porous^42^.

Lab experiments also show that adding anywhere from 1 to 4% nano-particles (NNPs) to the cement used decreases permeability (see Fig. 8). This occurs because the NNPs have a higher specific surface area than the replacement cement, allowing them to absorb more of the free water available during concrete mixing. The results also revealed that an increase in the W/C ratio increased permeability. This finding agrees very well with the literature^12,17,31–33,43^. Nevertheless, when NNP concentration was raised from 4 to 5%, an increase in WPD was seen. The high proportion might be explained by agglomeration, which is behind this growth^16^.

In terms of the MPS effect on the WPD, control concrete (the concrete without any nano-particles) exceeds all the concrete mixes, even in early curing days. This is because NNP plays a crucial role in quickening the hydration of cement particles via its filling action. Also, the use of NNPs with a smaller MPS caused a further decrease in the WPD of concrete. The smaller the MPS used, the better their filling properties will be.

### ML results

**Figure 9:**
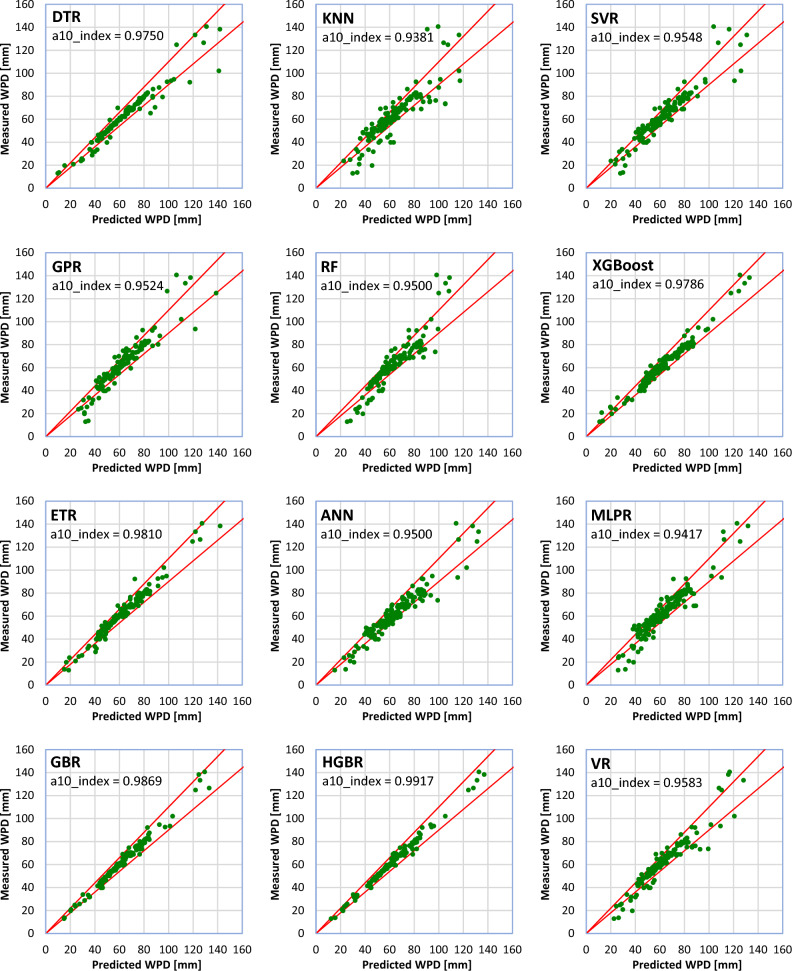
Comparison of the ML and laboratory test results.

At this stage, the performance of each of the ML methods is examined in estimating the WPD of different concrete samples compared to the laboratory tests. A comparison between the results estimated by each of the ML models and the results obtained from the laboratory tests is provided in Fig. 9 through the a10_index metric. As can be seen, all the ML models are in a geed agreement with the laboratory tests and have provided high accuracy in the estimation of the WPD, so the lowest accuracy is obtained by the KNN model (a10_index = 0.9381). Also, the highest accuracy is provided by the HGBR model (a10_index = 0.9917). Other models have provided accuracies between these two models.

**Figure 10:**
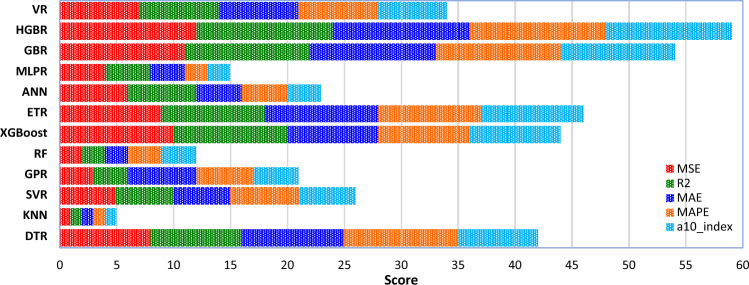
Ranking score of the ML methods' potential for the WDP estimation.

In addition to the a10_index, a wide range of additional statistical metrics (MSE, MAE, R^2^, and MAPE) are used to evaluate the performance of the ML models. Table 4 displays the models' respective statistical measures. In order to evaluate the effectiveness of the models, many statistical measures are used. At long last, the votes are tallied up, and each model has been given a ranking. The numerical positions are shown in Table 4. Table 4's data is graphically shown in Fig. 10. According to these results, the HGBR model is the most accurate, while the KNN model is the least accurate with ranking scores of 59 and 5, respectively. After evaluating each model's predictive accuracy, the following order was established: HGBR ‒ > GBR ‒ > ETR ‒ > XGBoost ‒ > DTR ‒ > VR ‒ > SVR ‒ > ANN ‒ > GPR ‒ > MLPR ‒ > RF ‒ > KNN. In fact, all the ML models have the necessary competence to estimate the WPD of NNP concrete due to the high accuracy they have provided. Therefore, these models can estimate the WPD of NNP concrete with high accuracy without the need to perform complex, time-consuming, and costly laboratory tests. Achieving such models is considered a very good development in the field of construction engineering. With the help of such models, it is possible to easily determine the composition of the desired concrete and to carry out the designs based on them as soon as possible.

**Table 4:** Assessment of the ML methods for the WPD of NNP concrete through scoring the statistical index results.

	MAP	Score	R^2^	Score	MAE	Score	MAPE	Score	a10_index	Score	Rank
DTR	29.63	8	0.9248	8	2.43	9	0.043	10	0.9750	7	42
KNN	94.58	1	0.7601	1	5.830	1	0.113	1	0.9381	1	5
SVR	41.74	5	0.8941	5	4.088	5	0.079	6	0.9548	5	26
GPR	46.28	3	0.8826	3	4.018	6	0.081	5	0.9524	4	21
RF	63.29	2	0.8394	2	4.972	2	0.096	3	0.9500	3	12
XGBoost	12.54	10	0.9682	10	2.552	8	0.045	8	0.9786	8	44
ETR	13.30	9	0.9663	9	2.322	10	0.044	9	0.9810	9	46
ANN	40.45	6	0.8974	6	4.727	4	0.084	4	0.9500	3	23
MLPR	43.74	4	0.889	4	4.742	3	0.097	2	0.9417	2	15
GBR	7.12	11	0.9819	11	1.592	11	0.026	11	0.9869	10	54
HGBR	4.93	12	0.9875	12	1.365	12	0.023	12	0.9917	11	59
VR	37.21	7	0.9056	7	3.552	7	0.069	7	0.9583	6	34

**Figure 11:**
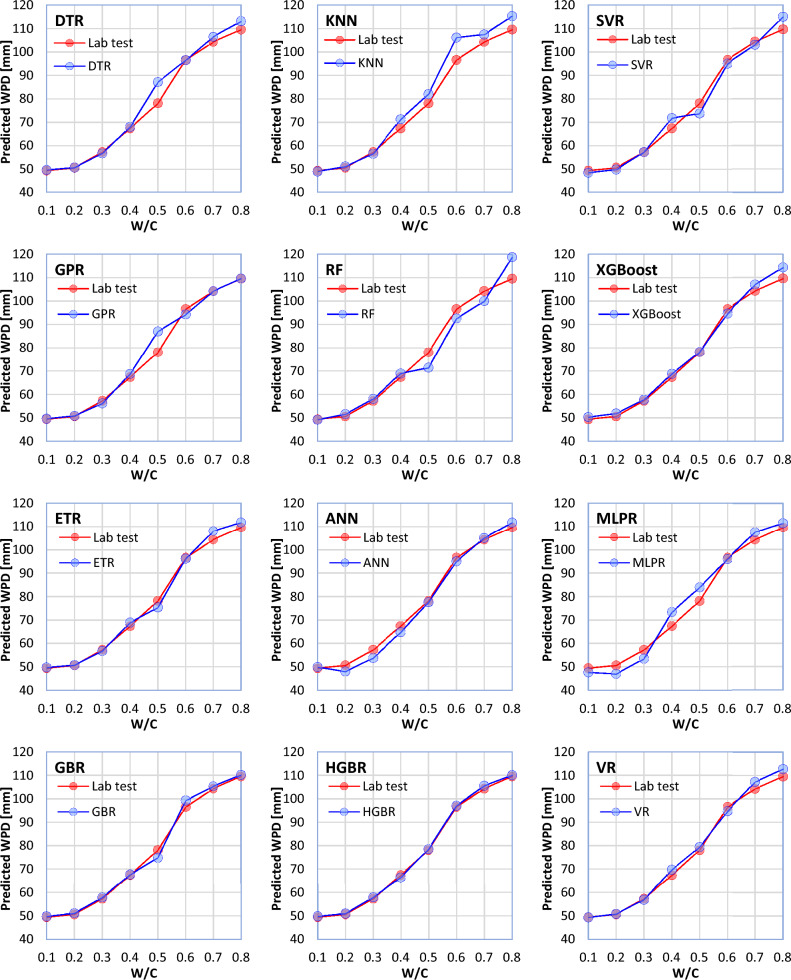
Behavior of the ML models about the parameter W/C (other input parameters are considered constant).

So far, a variety of ML models have been developed to estimate the WPD of NNP-reinforced concrete with remarkable prognostic accuracy. So, instead of merely looking at the cross-correlation, it would be preferable to examine input trends and output patterns to ensure generalization capabilities. This is done by maintaining a fixed set of certain inputs while altering others within a given range. Firstly, the performance of the models is examined by changing parameter W/C within its range (0.1, 0.2, 0.3, 0.4, 0.5, 0.6, 0.7, and 0.8) and maintaining other input parameters constant (CT = 7 days, NNP = 1%, MPS = 100 nm). Considering such conditions, 8 cubic concrete samples with dimensions of 15 × 15 × 15 cm were made for laboratory testing. According to the obtained laboratory results, by increasing the W/C ration and keeping other parameters constant, the WPD of concrete increases. Now, considering the eight concrete samples as the test data for the ML methods, it can be said that a model that shows more similar behavior to the laboratory test performs better. In Fig. 11, a comparison has been made between the laboratory results and the results obtained from the ML models for the eight concrete samples. According to Fig. 11, all the ML models have shown similar behavior to the laboratory tests. Therefore, it can be said that all the models present correct and acceptable behavior concerning the change in the W/C ratio. However, the most similar and most dissimilar behaviors compared to the laboratory tests are related to the HGBR and KNN models, respectively.

**Figure 12:**
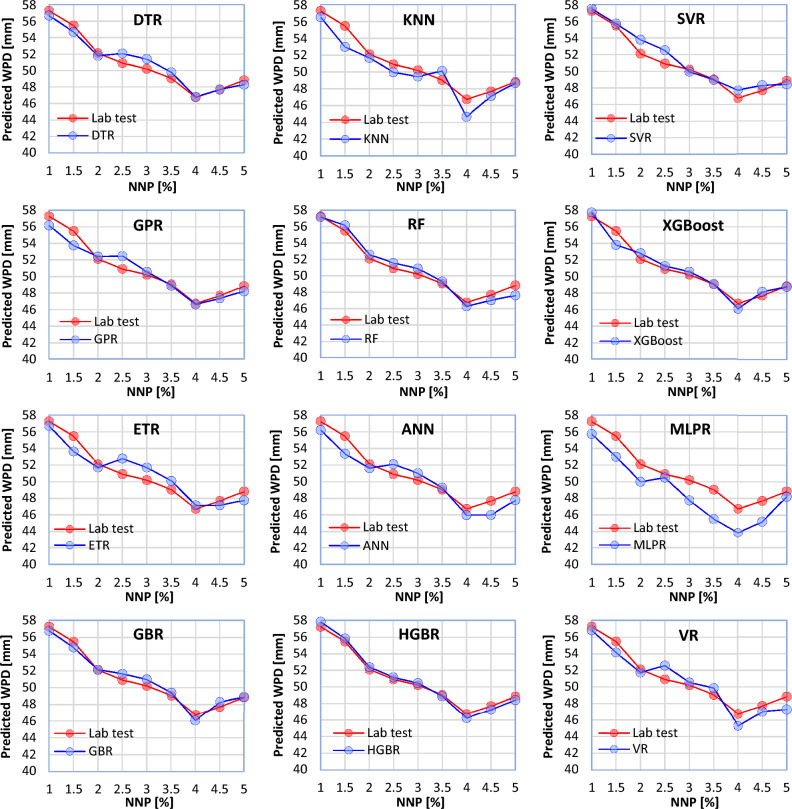
Behavior of the ML models in relation to the NNP content (other input parameters are considered constant).

Now the ML models' behavior is examined compared to the laboratory tests by changing the NNP content within its range (1%–5%) and keeping other parameters constant (CT = 7, W/C = 0.3, MPS = 100 nm). For this purpose, 9 cubic concrete samples with dimensions of 15 × 15 × 15 cm were made for laboratory testing. In making each of these samples, a different percentage of NNP was considered (1, 1.5, 2, 2.5, 3, 3.5, 4, 4.5, 5%). Laboratory results showed that by increasing the NNP content used in the concrete mixture from 1 to 4%, the WPD of the concrete decreases. Also, considering the NNP at more than 4% in the concrete mixture leads to an increase in the WPD. According to Fig. 12, the ML models have shown the same behavior concerning the change in the NNP content. In this case, all the ML models have presented very similar behavior to the laboratory tests. However, the HGBR and MLPR models have shown the best and worst performance, respectively.

**Figure 13:**
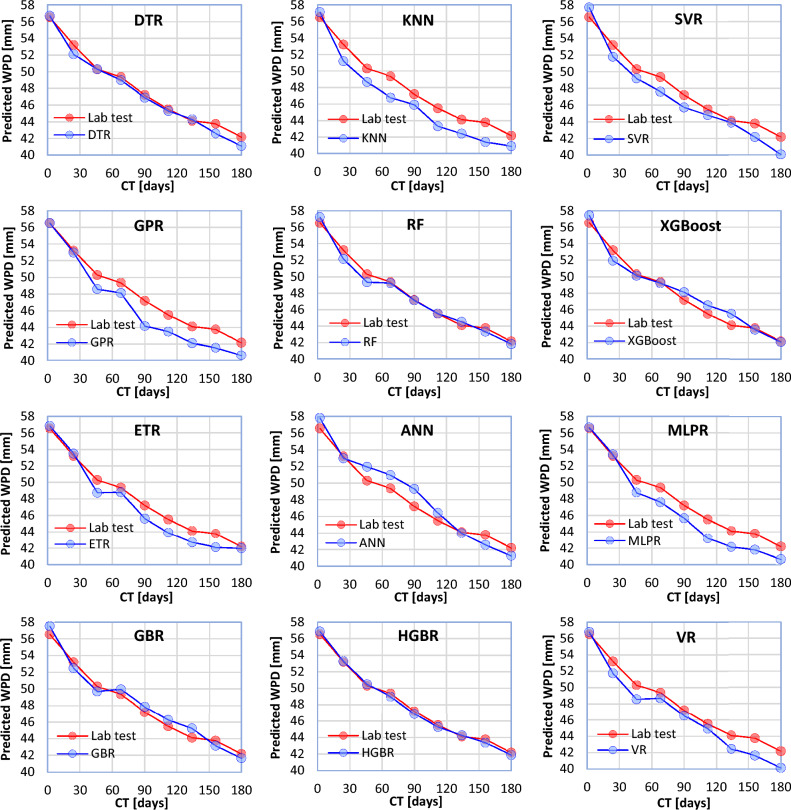
Behavior of the ML models in relation to the CT parameter (other input parameters are considered constant).

Laboratory results showed that increasing the CT decreases the WPD of concrete samples. In order to measure the behavior of each of the ML models with respect to the change of the CT, 9 samples with different CTs (2, 24, 46, 68, 90, 112, 134, 156, and 180 days) and their other characteristics fixed (NNP = 1%, W/C = 0.3, MPS = 100 nm) were subjected to laboratory tests. The data obtained from these samples was used as test data for the ML models. In Fig. 13, a comparison has been made between the behavior of each of the ML models and the behavior of the laboratory tests. As can be seen, all the models have shown similar behavior to the laboratory tests. However, the HGBR and KNN models have shown the best and worst performances compared to other models, respectively.

**Figure 14:**
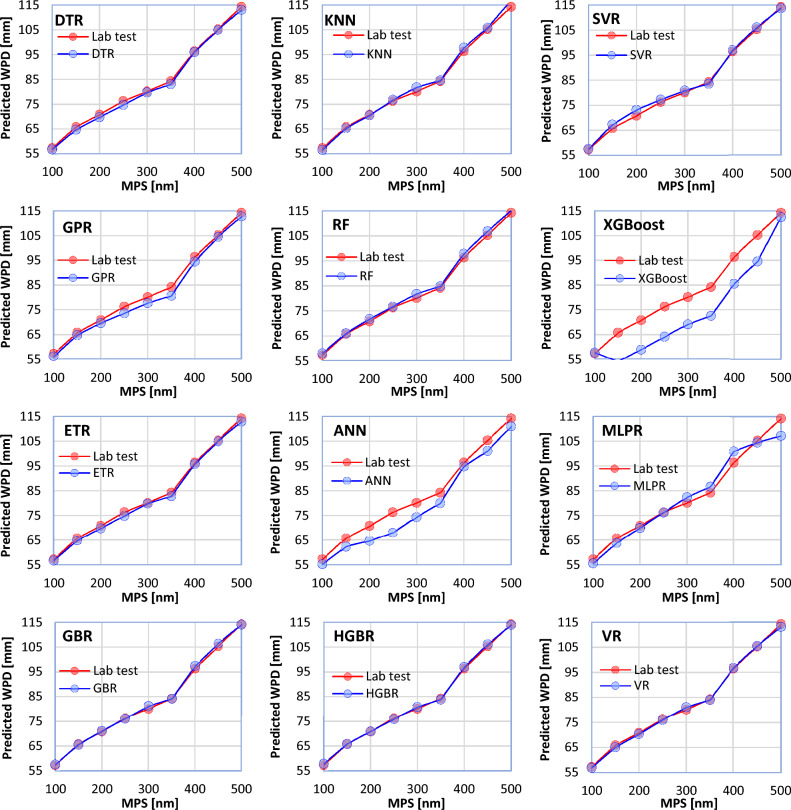
Behavior of the ML models in relation to the MPS parameter (other input parameters are considered constant).

Based on the results obtained from the laboratory tests, the use of smaller NNPs can lead to better filling of voids in the concrete structure, and as a result, it makes the concrete more impervious. To investigate the behavior of each of the ML models in relation to the MPS changes used in the production of concrete, nine concrete samples were prepared with different MPSs (100, 150, 200, 250, 300, 350, 400, 450, and 500 nm) of the NNP used in their mixture, and their other characteristics were fixed (CT = 7, W/C = 0.3, NNP = 1%). The ML results are compared with the laboratory results in Fig. 14. In this case, the correct performance of the models can also be confirmed. However, the most correct and incorrect results are related to the HGBR and KNN models, respectively.

**Figure 15:**
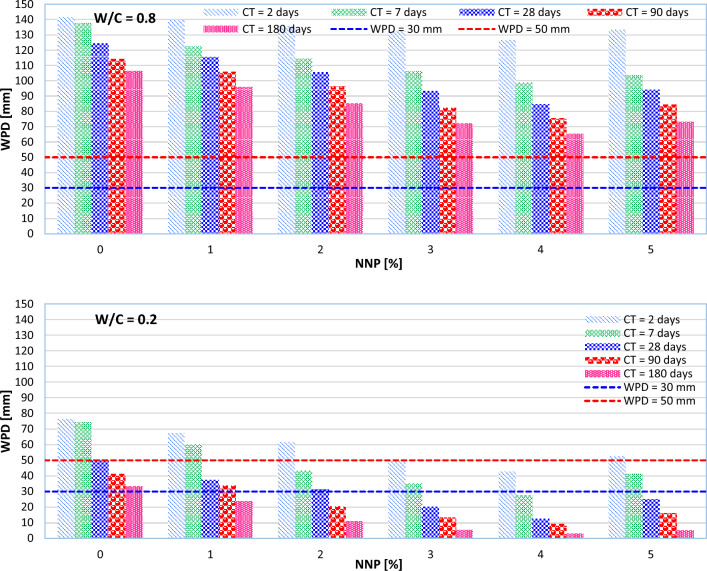
Investigating the behavior of the HGBR model with the simultaneous change of concrete characteristics.

The analysis of the results obtained up to this point showed that all the ML models used in this study have the potential to estimate the WPD of NNP-reinforced concrete. However, the highest accuracy was recorded for the HGBR model. Now, the ability of the HGBR model is examined more precisely to estimate the WPD of concrete. For this purpose, instead of only changing the value of one of the input parameters, in this case, simultaneously the values of all the input parameters are changed and the behavior of the HGBR model is recorded. Figure 15 shows the results recorded for the HGBR model. As can be seen, the results are very similar to the laboratory tests, so the WPD of concrete has decreased with the increase of the NNP content from 1 to 4%, the decrease of the W/C ratio and the MPS, and the increase of the CT. Such results were obtained exactly for the laboratory tests (see Fig. 8). Therefore, the ability of the HGBR model to estimate the WPD of the NNP-reinforced concrete can be confirmed, and this article presents this model as a suitable substitute for the time-consuming and expensive laboratory tests.

It should be noted that, ML-based models can only be used if the data needed to train them is really accessible. Otherwise, methods like laboratory tests and computer simulations we'd have are used. With the advent of the "data age," the groundwork is being laid for the development of more accurate ML-based models to estimate the WPD in concrete. Despite the fact that various papers have been published in this area, additional data is required to build ML models that are robust and adaptable enough for practical use. The WPD may be accurately predicted using ML models, as shown by these analyses. Yet, none of these models is perfect since it lacks completeness. That is, each investigation has taken into account its own unique set of factors. One of the most crucial factors to think about while creating new ML techniques is whether or not to enhance the database of previously presented models rather than change the kind of ML model compared to the earlier research. As a result, we may have a model that efficiently obtains the WPD for a variety of concrete kinds. It is not very useful to offer a model for one kind of concrete when it can only be used to predict the WPD of that type of concrete and not any others. A lot of effort remains before a model can be provided that can capture the WPD of most concretes under varying situations. Developing such an ML-based model needs a large database, which in turn necessitates international collaboration among academics.

### K-fold cross-validation

K-fold cross-validation is a technique used in machine learning to evaluate the performance and generalization ability of a model. It involves partitioning the dataset into K subsets of approximately equal size, commonly referred to as "folds." The model is trained K times, each time using K-1 folds as training data and the remaining fold as validation data. This process is repeated K times, with each fold used exactly once as the validation data.

**Figure 16:**
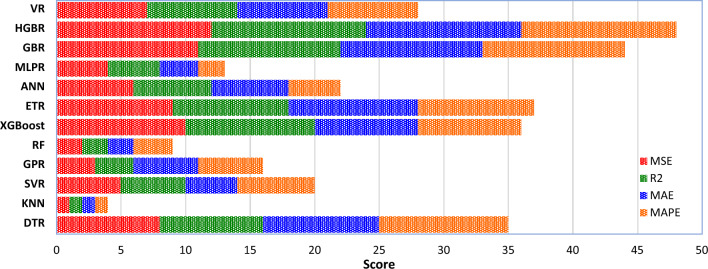
Ranking score of the ML methods' potential for the WDP estimation.

In this research, the K-fold cross-validation (K = 5) technique is also used to check the performance of the ML models with more confidence. The statistical evaluation criteria results are provided in Table 5. Table 5's data is graphically shown in Fig. 16. According to these results, still the HGBR model is the most accurate, while the KNN model is the least accurate with ranking scores of 48 and 4, respectively. After evaluating each model's predictive accuracy, the following order was established: HGBR ‒ > GBR ‒ > ETR ‒ > XGBoost ‒ > DTR ‒ > VR ‒ > ANN ‒ > SVR ‒ > GPR ‒ > MLPR ‒ > RF ‒ > KNN. In fact, all the ML models have the necessary competence to estimate the WPD of NNP concrete due to the high accuracy they have provided. Therefore, these results more reliably confirm the correct performance of the models.

**Table 5:** Assessment of the ML methods for the WPD of NNP concrete through scoring the statistical index results.

	MSE	Score	R^2^	Score	MAE	Score	MAPE	Score	Rank
DTR	33.7	8	0.9103	8	2.886	9	0.047	10	35
KNN	97.62	1	0.7422	1	5.974	1	0.116	1	4
SVR	45.81	5	0.8717	5	5.520	4	0.080	6	20
GPR	49.18	3	0.856	3	4.957	5	0.085	5	16
RF	68.05	2	0.8275	2	5.863	2	0.097	3	9
XGBoost	15.12	10	0.9516	10	2.977	8	0.052	8	36
ETR	15.73	9	0.9466	9	2.637	10	0.049	9	37
ANN	43.89	6	0.8863	6	4.885	6	0.088	4	22
MLPR	46.72	4	0.8681	4	5.703	3	0.102	2	13
GBR	8.17	11	0.9714	11	1.945	11	0.031	11	44
HGBR	5.41	12	0.9784	12	1.882	12	0.029	12	48
VR	35.37	7	0.8884	7	3.661	7	0.074	7	28

### Monte-Carlo uncertainty analysis

Monte Carlo uncertainty analysis is a powerful technique used to assess the uncertainty associated with mathematical models or simulations by generating multiple samples from probability distributions of input parameters. These samples are then used to propagate uncertainty through the model, yielding distributions of output variables. By repeatedly sampling input parameters and analyzing the resulting distributions of output variables, Monte-Carlo uncertainty analysis provides valuable insights into the variability and robustness of model predictions.

In this study, the Monte Carlo method was employed to ensure the accuracy of each algorithm. Through Monte Carlo simulation, each algorithm underwent 200 rounds of training and testing. During each iteration, 20% of the data were randomly allocated for testing purposes, while the remaining 80% were utilized for training. The accuracy achieved by each algorithm was recorded in each iteration, and the average accuracy was computed based on these results, as outlined in Table 6 for each algorithm. Remarkably, all algorithms demonstrated high accuracy levels. Specifically, the HGBR algorithm exhibited the highest accuracy at 0.95, while the KNN algorithm demonstrated the lowest accuracy at 0.78.

**Table 6:** The average accuracies of machine learning algorithms obtained from the Monte Carlo method.

HGBR	GBR	ETR	XGBoost	DTR	VR	ANN	SVR	GPR	MLPR	RF	KNN
0.95	0.92	0.90	0.89	0.87	0.85	0.85	0.83	0.81	0.81	0.80	0.78

### Sensitivity analysis

Parameter sensitivity analysis is another problem that consistently arises in ML-based models. Sensitivity analysis is utilized to determine which model factors had the most influence. A solid understanding of this topic is crucial for reducing data dimensionality and avoiding model overfitting. In this research, the mutual information (MI) technique was used for the sensitivity analysis.

With its roots in information theory, MI applies the concept of information gain (often used in the creation of decision trees) to the problem of feature selection. MI between two variables measures the amount that can be inferred about one variable from observations of the other. When dealing with categorical inputs and outputs, MI's implementation becomes a breeze. It was originally designed to deal with textual data, but it may be adapted to deal with numerical data as well. The degree to which MI has been able to reduce entropy is a measure of its effectiveness. Let's use Eq. 7 as an example to get a feel for the concept. A positive number between zero and infinity should be used for the MI score. Increase the importance of a feature in the model-training process if its MI value is high. Nevertheless, if the MI score is very low, such as 0, then the connection between the trait and the goal is shaky at best.

7 $$MI\left(feature;target\right)= Entropy\left(feature\right)- Entropy(feature|target)$$

**Figure 17:**
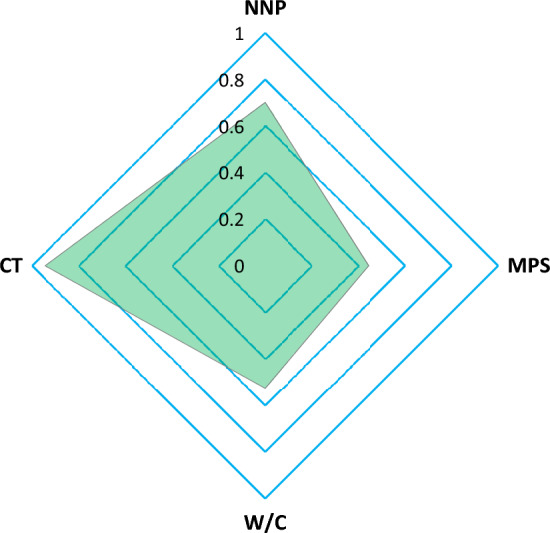
The input variables' relative relevance scores for the examined output (WPD).

The relative weights of the input features (NNP, CT, W/C ratio, and MPS) used to estimate WPD are shown in Fig. 17. As seen in Fig. 17, all of these factors influence the WPD of concrete in some way. A MI score of 0.945 indicated that CT was the most important factor. The MI scores of 0.701, 0.527, and 0.444 indicate that other factors, such as the NNP, W/C ratio, and MPS of the NNP, also significantly affect the concrete's WPD.

### Graphical user interface

**Figure 18:**
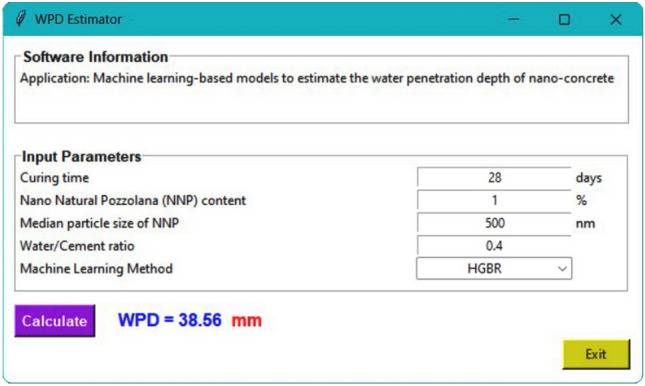
GUI of the ML‒based models for estimating concrete’s WPD.

Concrete's WPD may be estimated with the use of graphical user interfaces (GUIs), which are user-friendly tools that may give ML-based estimation models. In this study, all twelve ML algorithms are included in the program. There is no need to retrain the ML models for the estimation of the WPD since they were already trained on the laboratory dataset. The user interface (Fig. 18) is polished and intuitive. When the user enters the input parameters into the GUI, the trained ML algorithms may estimate the output parameter (WPD). As compared to other approaches, the GUI can estimate the concrete's WPD with good accuracy in a minimum amount of time and at the lowest possible cost. In addition, it has the potential to serve as a study platform for those interested in amassing data on the WPD of concrete. Each ML model had a different amount of time it took to compute, from a low of 2 s (KNN) to a high of 6.37 min (RF).

### Key limitations of the work

The ML models presented in this study are only able to estimate the WPD of those concrete samples that have some NNP used in their production. In order to be able to investigate the effect of different types of nanoparticle additives on the WPD of concrete with the help of these models, the database used in this study needs to be expanded. For example, an input parameter called the type of nanoparticles used in the concrete mixture should be used in the database. In this way, the application of the models proposed in this study can be expanded.

## Conclusions

### Results

1. The values estimated by the ML models were not far from the laboratory tests. Statistical metrics, such as R^2^, MAE, MSE, a10_index, and MAPE demonstrated that all the twelve ML models proposed in this study can accurately estimate the WPD of NNP-reinforced concrete. However, the most and least robust estimates were produced by the HGBR and KNN models, respectively. Therefore, ML-based models can be a practical replacement for other costly and time-consuming laboratory methods for determining the WPD of concrete.
2. Based on the comparison of the results obtained from the ML models with the laboratory results, the models were arranged in terms of estimation accuracy as follows: HGBR ‒ > GBR ‒ > ETR ‒ > XGBoost ‒ > DTR ‒ > VR ‒ > SVR ‒ > ANN ‒ > GPR ‒ > MLPR ‒ > RF ‒ > KNN.
3. Based on both the laboratory and ML results, an NNP content of 4% appears to be the ideal dosage, for which the WPD values were the lowest.
4. By comparing the ML models' behavior with the laboratory tests by changing the value of one of the input parameters and keeping the values of the other parameters constant, it was concluded that the HGBR model works more correctly than the other models and therefore was suggested as the most suitable model for the concrete’s WPD estimation.
5. All of the investigated factors (NNP content, CT, MPS, and W/C ratio) were shown to have a significant influence on the WPD of the concrete using a sensitivity analysis. Nevertheless, the MI score of 0.945 indicates that CT is the most important factor. The MI scores of 0.701, 0.527, and 0.444 indicate that other factors, such as the NNP, W/C ratio, and MPS of the NNP, also significantly affect the concrete's WPD.
6. To further aid in the estimation of concrete’s WPD for engineering challenges, a GUI software for the ML-based models was developed.
7. The suggested ML models may be effectively employed in the management of concrete quality because they may give fresh information or experiences that contribute to a better understanding of the complex nature of the interaction between the ingredients and the qualities of NNP-reinforced concrete. In addition, the site engineers may benefit from such knowledge or experience when attempting to estimate the concrete's quality in advance of placement based on the mix's components, the amount of time required to open the formwork, and other factors unique to the project. Additionally, NNP might be useful in the design of such a concrete mix.

### Key limitations and suggestions

The ML models outlined in this study are specifically tailored to predict the WPD of concrete samples incorporating NNP. To extend the applicability of these models and explore the impact of various nanoparticle additives on concrete WPD, the dataset utilized in this research requires expansion. For instance, incorporating an input parameter specifying the type of nanoparticles integrated into the concrete mixture would enhance the database. By including this additional variable, the models proposed in this study can be applied to investigate a broader range of nanoparticle additives and their effects on concrete permeability. Therefore, to verify the findings generated by the established models, it is strongly advised that more research be conducted, integrating additional variables and using a bigger dataset. NP chemical composition, nanoparticle type, various durability-related qualities, and varying NNP fineness levels are all variables that may be explored further. So, more information will be at hand, allowing for a more precise estimate. Expanding the dataset in this manner would not only broaden the scope of the study but also increase the versatility and utility of the ML models, facilitating their application across diverse nanoparticle-enhanced concrete formulations.

## Data Availability

The data and the GUI that support the findings of this study are available from corresponding author (Dr. Sivaprakasam Palani) but restrictions apply to the availability of these data, which were used under license for the current study, and so are not publicly available. Data and the GUI are however available from the authors upon reasonable request and with permission of the corresponding author (Dr. Sivaprakasam Palani).
